# Exploring Specific miRNA-mRNA Axes With Relationship to Taxanes-Resistance in Breast Cancer

**DOI:** 10.3389/fonc.2020.01397

**Published:** 2020-08-21

**Authors:** Danni Chen, Chang Bao, Feng Zhao, Haogang Yu, Guansheng Zhong, Liang Xu, Senxiang Yan

**Affiliations:** ^1^Department of Radiation Oncology, The First Affiliated Hospital, College of Medicine, Zhejiang University, Hangzhou, China; ^2^Program of Innovative Cancer Therapeutics, Division of Hepatobiliary and Pancreatic Surgery, Department of Surgery, The First Affiliated Hospital, College of Medicine, Zhejiang University, Hangzhou, China; ^3^Key Laboratory of Organ Transplantation, the First Affiliated Hospital, College of Medicine, Zhejiang University, Hangzhou, China; ^4^Key Laboratory of Combined Multi-organ Transplantation, Ministry of Public Health, Hangzhou, China; ^5^Breast Center, The First Affiliated Hospital, College of Medicine, Zhejiang University, Hangzhou, China; ^6^Clinical Research Center, The First Affiliated Hospital, College of Medicine, Zhejiang University, Hangzhou, China

**Keywords:** breast cancer, taxanes-resistance, miR-335-5p/CXCL9 axis, let-7c-5p/CCR7/SOCS1 axis, WCGNA

## Abstract

Breast cancer is the most prevalent type of malignancy in women worldwide. Taxanes (paclitaxel and docetaxel) are widely applied as first-line chemotherapeutic agents, while the therapeutic effect is seriously limited by the development of drug resistance. In the present study, we screened out several miRNAs dysregulated in taxanes-resistant breast cancer samples and confirmed that two miRNAs (miR-335-5p and let-7c-5p) played a major role in cell proliferation, apoptosis, and chemo-resistance. In addition, the weighted gene co-expression network analysis (WGCNA) for potential target genes of miR-335-5p and let-7c-5p identified three hub genes (CXCL9, CCR7, and SOCS1) with a positive relationship to taxanes-sensitivity. Further, target relationships between miR-335-5p and CXCL9, let-7c-5p and CCR7/SOCS1 were confirmed by dual-luciferase reporter assays. Importantly, the regulatory functions of CXCL9, CCR7, and SOCS1 on proliferation and chemoresistance were validated. In conclusion, our study shed light on clinical theragnostic relationships between miR-335-5p/CXCL9, let-7c-5p/CCR7/SOCS1 axes, and taxanes-resistance in breast cancer.

## Introduction

Breast cancer is the most prevalent type of malignancy in women worldwide (1). In developed countries, the incidence rate of breast cancer among young women increases rapidly, which is due partly to earlier diagnosis by the development of detection technology. While in developing countries, the strikingly higher incidence and mortality rates may be mainly ascribed to increasing populations, and improper healthcare (2). Currently, several treatment modalities are available to defend against breast cancer, including surgical resection, chemotherapy, radiotherapy, endocrinotherapy, targeted therapy, and immunotherapy (3). As a cornerstone of therapeutic strategies, chemotherapy is widely applied both in adjuvant and neoadjuvant settings, and can be used alone or in combination with other treatments (4).

There are multitudinous chemotherapeutic agents commonly applied in clinics, such as taxanes (e.g., paclitaxel, docetaxel), anthracyclines (e.g., doxorubicin, epirubicin), carboplatin, alkylating agents (e.g., cyclophosphamide), and anti-metabolites (e.g., 5-fluorouracil). However, a successful long-term effect of chemotherapeutic agents is vastly limited by the development of drug resistance (5). The drug resistance, mainly including intrinsic and acquired resistance, is an intricate phenomenon induced by multiple genetic and epigenetic factors (6). Acquired chemo-resistance is relatively more common in clinics, which means tumors initially sensitive to chemotherapeutic agents become more and more insensitive during the course of treatment (7). What's worse, once tumors manifest resistance to a specific drug, they often resist to a large-scale of other chemotherapeutic agents that are chemically and functionally unrelated. This phenomenon is summarized as multidrug resistance (MDR) (8).

Taxanes belong to Tetracyclic diterpenes and are originally isolated from the bark of Taxus Chinensis. Since the 1990's, taxanes have been commonly applied as the first-line therapy for breast cancer, mainly including paclitaxel (Taxol^®^) and docetaxel (Taxotere^®^) (9, 10). The main cytotoxicity of taxanes is considered to promote tumor cells apoptosis and blockade cell proliferation by binding to beta-tubulin and microtubule-stabilizing agents (11, 12). Unfortunately, the effectiveness of taxanes is also limited by the development of chemo-resistance, including MDR (13). In the past two decades, researchers have focused on figuring out the major roles of mediated taxanes-resistance. Cytochrome P450 isoenzymes (CYPs) system was reported to play an important role in drug metabolism, and aberrant expression of CYPs may cause metabolic inactivation of paclitaxel, tamoxifen (14). ABCB1 encodes Multiple Resistance protein (MDR1), which involved in the cellular efflux of chemotherapeutic drugs. High expression and fusion-positive of ABCB1 increased active efflux of paclitaxel, doxorubicin, inducing resistance of multiple drugs (15, 16). In addition, dysfunction of microtubules, both α and β tubulin, disordered cell cycle regulation, and altered cellular apoptosis are also viewed as important mechanisms in taxanes-resistance (17–20). Because of the attribution of multifaceted cellular dysfunctions, the interventions to reverse taxanes-resistance are far from being practicable.

Due to the almost inevitable development of taxanes-resistance in breast cancer, it is urgently needed to explore accurate biomarkers for the prediction and reverse of the resistance. The discovery of microRNAs (miRNAs) brought lightness for these two purposes. MiRNAs are a group of small, non-coding RNAs, consisting of 19–25 nucleotides (~22 nt) in a single-strand. Most miRNAs regulate its target genes expression through modulation of post-transcriptional activity, by binding to the 3′-UTR of multiple target mRNAs in a sequence-specific manner (21, 22). Numerous miRNAs have been shown to regulate diverse biological activities and dysregulation of miRNAs always plays a crucial role in tumorigenesis (23, 24). In addition, plenty of researches has been conducted and highlighted the role of miRNAs in chemo-resistance (25–27). Several clinical trials were completed, which strongly encouraged the potential use of miRNAs-based treatments in clinical practice. MRG106, an anti-miR-155, has been evaluated in phase I for safety, tolerability and efficacy in treating mycosis fungoides (a type of cutaneous T-cell lymphoma). The results showed MRG-106 is well-tolerated and has high clinical activity and potential for a better quality of life. The encouraging data promoted phase II clinical trial of MRG-106 in other hematological malignancies, including chronic lymphocytic leukemia (CLL), Diffuse Large B-Cell Lymphoma (DLBCL) and adult T-cell leukemia/lymphoma (ATLL) (28). In addition, a phase I study suggested acceptable safety and high clinical activity of miR-16 mimic (TargomiR) in patients with malignant pleural mesothelioma (MPM) and advanced non-small cell lung cancer (NSCLC). Subsequent trials were encouraged for combinational treatment of TargomiR with chemotherapy and immune checkpoint inhibitors (29). Growing evidence on the dramatic importance of miRNA-target mRNA pairs in taxanes-resistance have been confirmed [e.g., miR-34a-NOTCH1 axis (30), miR-125b-Sema4C axis (31), miR-503-CD97-JAK2/STAT3 pathway (32)]. However, most studies emphasized the effect of single miRNA and target genes in the process, which may ignore the integrated function of all dysregulated miRNA-mRNA pairs.

In the present study, we screened out several dysregulated miRNA-mRNA axes relevant to taxanes-resistance, and confirmed the regulation of miR-335-5p and let-7c-5p on diverse biological activities, including cell proliferation, apoptosis, and taxanes-resistance. Weighted gene co-expression network analysis (WGCNA) was carried out for potential target genes of miR-335-5p and let-7c-5p. The hub genes (CXCL9, CCR7, and SOCS1) in the downstream of these two miRNAs were identified and confirmed with close relationship to taxanes-resistance. We anticipate the study could help to better understand miRNA-mRNA axes involved in taxances-resistance and potentially serve as a reverse of the drug resistance.

## Materials and Methods

### Screening for Dysregulated miRNAs Relevant to Taxanes-Resistance

For patient analysis, all data were retrieved from the TCGA data portal (https://genome-cancer.ucsc.edu/) updated by the end of December 31, 2018. There were 95 breast cancer patients who had received chemotherapy including taxanes (Table S5). The clinical efficacy was evaluated according to Response Evaluation Criteria in Solid Tumors (RECIST). Patients received complete response (CR) and partial response (PR) were divided into taxanes-sensitive group (*n* = 79), while patients received progressive disease (PD) and stable disease (SD) were divided into taxanes-resistant group (*n* = 16). The miRNA expressions were measured using IIIumina GASeq/HiSeq 2000 Sequencing, normalized and calculated via R package edgeR (3.30.0) from the Bioconductor project. *P* < 0.05 and |log2 fold change (log2FC) |>1 was set as thresholds for dysregulated miRNAs relevant to taxanes-resistance.

### Weighted Gene Co-expression Analysis (WGCNA)

A co-expression network was built via R package WGCNA from the bioconductor project. Briefly, we assessed the common DEGs of a cluster of samples to check the validation. Then, soft threshold power β was validated from scale independence and mean connectivity in accordance with standard scale-free networks (Figures S1A–E). Furthermore, we calculated the association between filtered genes to form a topological overlap matrix (TOM). Based on dissimilarity calculated in TOM, average linkage hierarchical clustering was classified for cluster dendrogram. Moreover, the dissimilarity of module eigengenes (ME) was assessed and similar modules were merged with a height cutoff as 0.20. ME was defined as the core of a module and represented the genes expression profiles. The association of ME expression and clinical characteristics was calculated to define the relevant module. Gene significance (GS) was defined as log_10_ (*P*-value) in the linear regression, and module significance (MS) represented average GS of all the genes in the module.

### Cell Transfection

The mature miRNA mimic, miRNA inhibitor and NC control were purchased from Ribobio, Guangzhou, China (Table S2), and the plasmids overexpressing of CXCL9, CCR7, and SOCS1 were purchased from HANBIO, Shanghai, China. The above reagents were transfected into cells by using Lipofectamine™ 3000 according to the manufacturer's instruction. After 12 h, the cell culture medium was changed. The transfection effects were validated by qRT-PCR and Western Blot (Figures S2C–E). The influence on cell proligeration and taxanes-resistance by NC mimics/inhibitors and NC control of blank plasmids were little small at appropriate concentration (Figure S3).

### Edu Incorporation Assay

For Edu incorporation assay, transfected cells were harvested, resuspended to a final concentration of 1 × 10^4^ cells/mL, and distributed into 24-well plates. After 12 h transfection, cells were cultured in fresh medium for 96 h. Five-ethynyl-2'-deoxyuridine (Edu) incorporation assays were carried out using the Cell-Light™ Edu imaging detecting kit according to the manufacturer's instructions (RiboBio, Guangzhou, China).

### Colony Formation Assay

Cells were harvested, resuspended, and distributed into 6-well plates. After 12 h transfection, the cell culture medium was replaced by fresh medium every 2 days. The cells were re-transfected on the 6th day. After another 6 days, cells were fixed with 4% paraformaldehyde and dyed with 0.1% crystal violet staining solution. The visible colonies with more than 50 cells under the microscope were calculated.

### Apoptosis Analysis

Cells were harvested, resuspended, and distributed into 6-well plates (2 × 10^5^ cells per well). After 12 h transfection, cells were cultured in medium with PTX (200 or 10 nM) and non-PTX for 72 h. AnnexinV/PI apoptosis detection kit (Beyotime, Haimen, China) was used to detect cell apoptosis according to the manufacturer's instructions, and the percentage of apoptotic cells was determined by flow cytometry using a BD FACSCalibur™ flow cytometry system.

### Cell Viability Assay

Cells were harvested, resuspended, and distributed into 96-well plates. After 12 h transfection, the designated columns were treated with paclitaxel or docetaxel for 72 h. Then, 3-(4,5-dimethylthiazol-2-yl)-2,5 diphenyltetrazolium bromide (MTT) assays were performed to detect cell viability. “Relative cell viability” = the viability of cells in drug-containing medium / the viability of cells in drug-free medium. “Relative cell viability” was further fitted to a dose-response curve to estimate the IC_50_ by the Graphpad Prism 6 software.

### Dual-Luciferase Reporter Assay

The 3' UTRs of CXCL9, CCR7, and SOCS1 containing upstream miRNAs putative target sites (Figure S2A) were amplified and cloned into psiCHECK-2 (Promega). A Fast Mutagenesis kit (VazymeBioTech) was used to mutate the binding sites as Figure S2B according to the manufacturer's instructions (Table S3). Dual-luciferase assays were performed using 1 × 10^4^ MCF-7 cells per well in a 96-well plate. Following attachment xs. for 12 h, the cells were co-transfected with 50 ng respective reporter constructs with either NC mimics or miRNA mimics (50 nM). After 48 h, the Reporter Assay System Kit (Promega) was used to measure the luciferase activity. Firefly luciferase activity was normalized to constitutive renilla luciferase activity.

## Results

### Upregulation of miR-335-5p, Let-7c-5p, and miR-99a-5p Were Correlated With Taxanes-Resistance and Poor Prognosis in Breast Cancer

**Figure 1:**
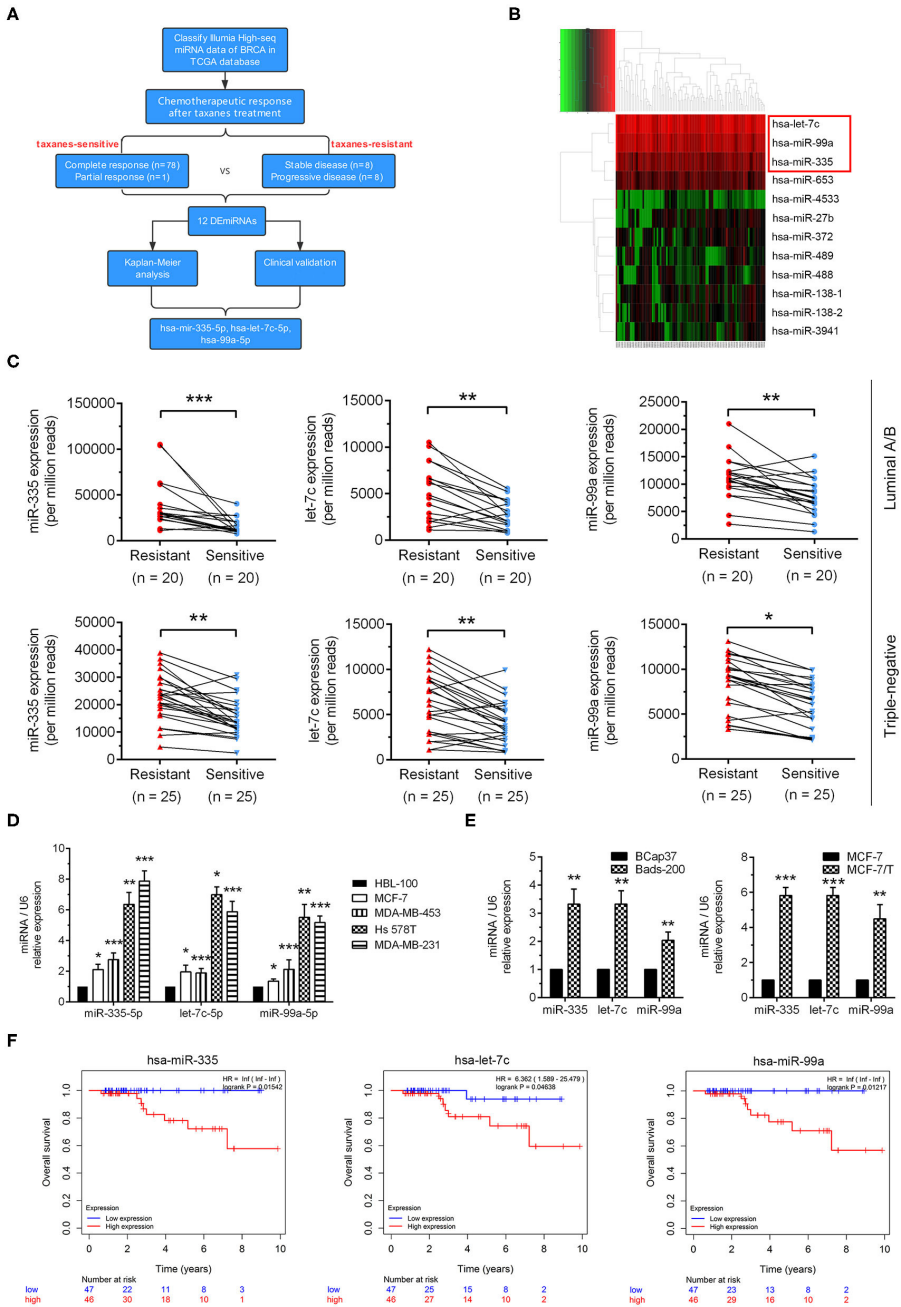
Identification of dysregulated miRNAs relevant to taxanes-resistance in breast cancer. **(A)** The workflow of identification of miRNAs relevant to taxanes-resistance in breast cancer. **(B)** The heatmap showed dysregulated miRNAs in tumor tissues from comparation of taxanes-resistant and taxanes-sensitive groups. **(C)** Three selected miRNAs (miR-335-5p, let-7c-5p, and miR-99a-5p) were confirmed upregulation in tumor samples of taxanes-resistant breast cancer patients. **(D)** miR-335-5p, let-7c-5p, and miR-99a-5p were upregulated in breast cancer cell lines compared with breast cell line. **(E)** Three selected miRNAs were upregulated in paclitaxel-resistant breast cancer cell lines. **(F)** High expression of selected miRNAs predicted poorer overall survival in patients with breast cancer in the TCGA database. ^*^P < 0.05, ^**^P < 0.01, ^***^P < 0.001.

As the workflow shown in Figure 1A, Illumia High-seq miRNA data of breast cancer samples were initially downloaded from TCGA database. According to Response Evaluation Criteria in Solid Tumors (RECIST), patients received taxanes treatments were divided into taxanes-sensitive (*n* = 79) group and taxanes-resistant group (*n* = 16) (Table S5). To determine the role of miRNAs in taxanes-resistance, miRNAs expressions in tumor tissues were normalized via edgeR package and compared between the above groups. Eleven miRNAs were significantly increased in taxanes-resistant group, while miR-27b was low expression (Figure 1B). Afterwards, we tested the above dysregulated miRNAs in breast cancer tissues in clinic. According to efficacy evaluation outcomes, patients were divided into taxanes-resistant/-sensitive groups (Supplementary Materials and Methods, Table S4). The results of qRT-PCR indicated that miR-335-5p, let-7c-5p, miR-99a-5p were markedly upregulated in taxanes-resistant group both in luminal and triple-negative subtypes (Figure 1C). Next, we determined miRNA expressions in breast cell line (HBL-100) and a panel of breast cancer cell lines. MiR-335-5p, let-7c-5p, miR-99a-5p were upregulated in four tested cell lines, particularly in Hs 578T and MDA-MB-231 cells (Figure 1D). Intriguingly, similar results were observed in two established paclitaxel-resistant sublines (MCF-7/T and Bads-200) compared with parental cell lines (MCF-7 and BCap37) (Figure 1E). Moreover, among the selected miRNAs, high expressions of miRNAs (miR-335-5p, let-7c-5p, miR-99a-5p) were associated with poor prognosis of breast cancer patients in the TCGA database (*n* = 93) (Figure 1F, Table S5). These results demonstrated that miR-335-5p, let-7c-5p, miR-99a-5p were upregulated in breast cancer samples in taxanes-resistant group and multiple breast cancer cell lines, suggesting a pro-tumorigenic role in taxanes-resistance.

### Knockdown of miR-335-5p, Let-7c-5p Inhibited Cell Proliferation, and Reversed Taxanes-Resistance in Breast Cancer Cells

**Figure 2:**
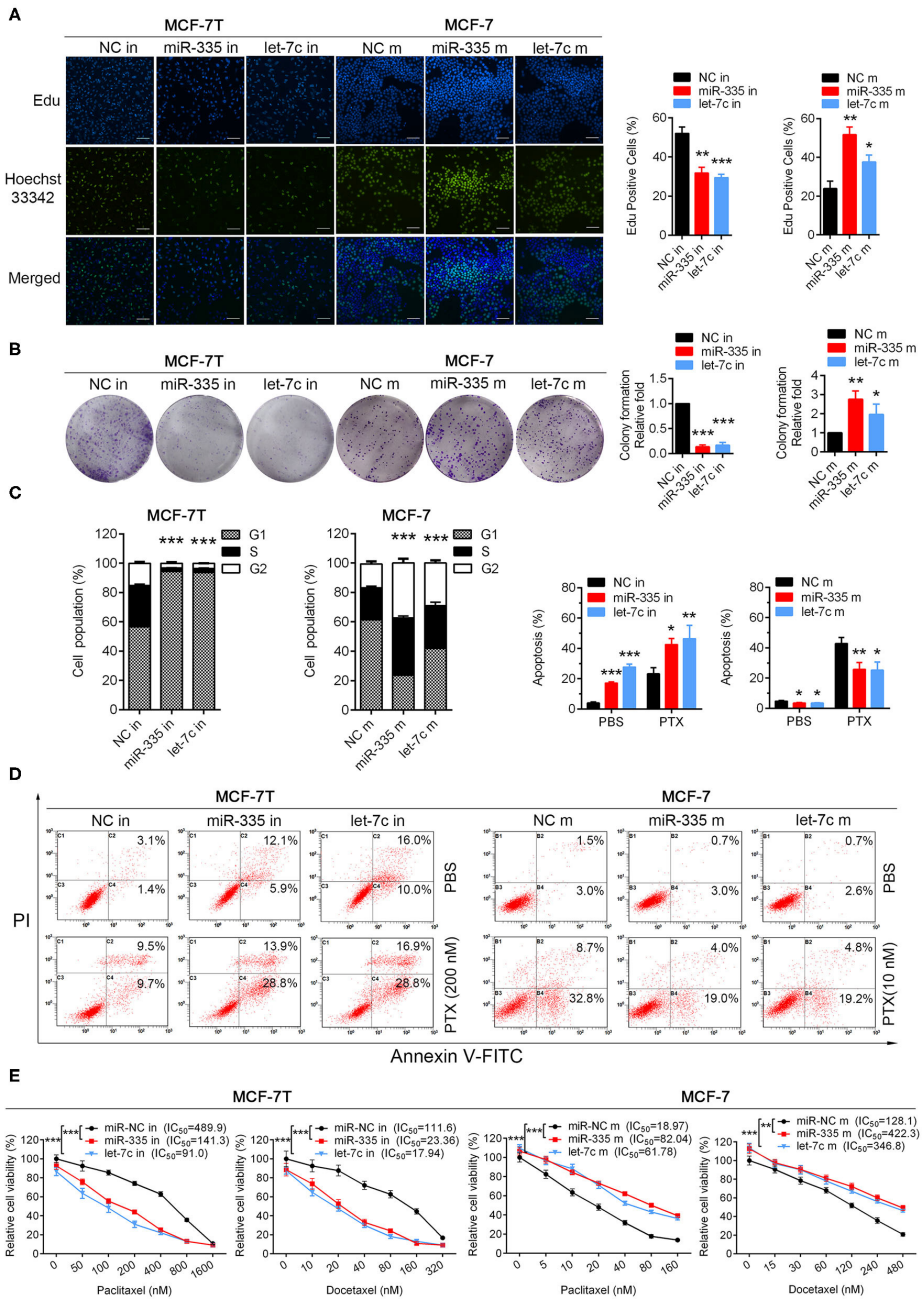
Validation biological functions of selected miRNAs in paclitaxel-resistant/sensitive cells. **(A)** Edu incorporation assays, **(B)** colony formation assays, **(C)** cell cycle assays were performed both in MCF-7/T and MCF-7 cells. **(D)** Apoptosis rate was detected in miR-335-5p/let-7c-5p-overexpressing cells and miR-335-5p/let-7c-5p-decreasing cells with or without paclitaxel. **(E)** The IC_50_ to paclitaxel and docetaxel was detected by MTT assays. All experiments were performed at least three times independently. The data were presented as the mean ± *SD*. ^*^P < 0.05, ^**^P < 0.01, ^***^P < 0.001. Scale bar: 500 μM.

To determine the biological function of miR-335-5p, let-7c-5p, and miR-99a-5p in breast cancer cells, we performed both loss- and gain-of function studies in paclitaxel resistant cell line (MCF-7/T) and parental cell line (MCF-7) Figure S2C. Knockdown of miR-335-5p and let-7c-5p in MCF-7/T cells suppressed cell growth/proliferation, respectively in Edu incorporation, colony formation and cell cycle assays (Figures 2A–C). In addition, miR-335-5p and let-7c-5p knockdown also increased cell apoptosis rate, especially promoted paclitaxel-induced cell apoptosis (Figure 2D). Down-expression of miR-335-5p and let-7c-5p significantly reversed cell taxanes-resistance, by decreasing paclitaxel and docetaxel IC_50_ of MCF-7/T cells via MTT assays (Figure 2E). Overexpression of miR-335-5p and let-7c-5p in MCF-7 cells markedly increased cell proliferation, reduced cell apoptosis and sensitivity to paclitaxel and docetaxel (Figures 2A–E). These findings suggest that miR-335-5p and let-7c-5p are required for the taxanes-resistance of breast cancer cells. However, we didn't observe similar role of miR-99a-5p in loss- and gain-of function studies.

### Key Gene Module Relevant to Taxanes-Sensitivity was Identified via WGCNA

**Figure 3:**
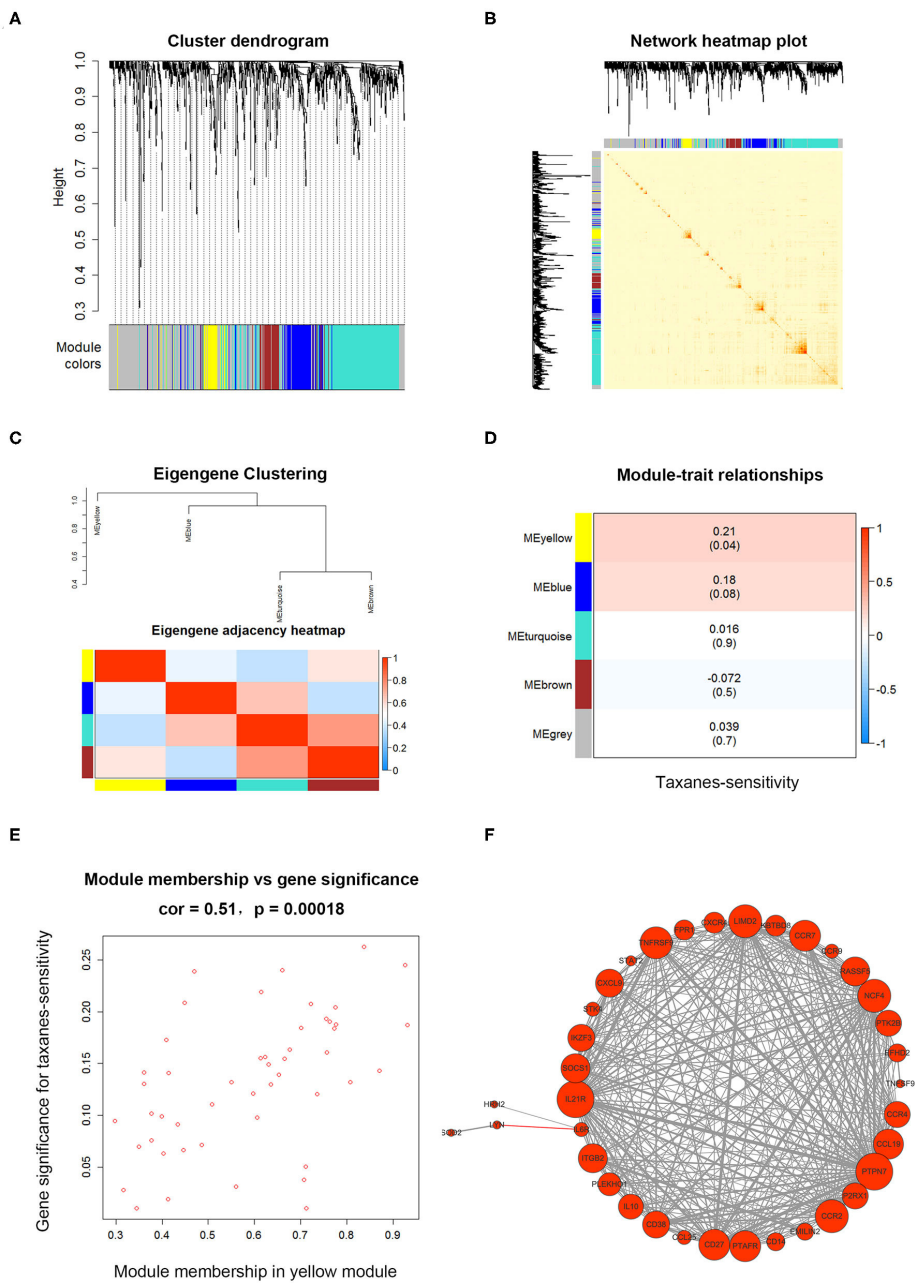
The WGCNA for candidate target genes of miR-335-5p and let-7c-5p. **(A)** Clustering dendrogram of target genes of miR-335-5p and let-7c-5p, with dissimilarity based on Topological Overlap Measure (TOM). Genes were divided into five assigned merged modules, including 350 genes in gray module represented not co-expressed. **(B)** Interactive relationship analysis of co-expression genes in different modules. The brightness of yellow in the middle suggested a high-scale independence among the modules. **(C)** Eigengene adjacency heatmap showed the calculated eigengene of each module yielded in the above clustering analysis. **(D)** Module-trait associations between eigengenes in the module and taxanes-sensitivity of based on TCGA database. The yellow module showed highest correlation. **(E)** A scatterplot of Gene Significance (GS) and module membership (MM) in yellow module. There is a significantly positive correlation between GS and MM in the module. **(F)** Weight coefficient was calculated between pairwise genes in the yellow module and the hub genes were presented as different sizes of circles according to weight coefficient.

We next used candidate and unbiased screening approaches to determine the potential mRNA targets of miR-335-5p and let-7c-5p in promoting taxanes-resistance function. First, we started with a candidate approach, predicting on potential target genes of miRNAs by the miRNet database (Table S6). Then the weighted gene co-expression analysis (WGCNA) was conducted to further clarify the target genes with relationship to taxanes-sensitivity. Firstly, the input dataset for WGCNA construction consists of 908 candidate target gene expressions in 95 breast cancer tumor samples. After quality assessment, the power of β = 6 (scale-free *R*^2^ = 0.9) was selected to construct a scale-free network. After that we set MEDissThres as 0.20 to merge similar modules, and a total of five colored modules were classified via Topological Overlap Measure (TOM) (Figure 3A). Then network heatmap was performed and the results showed the independence between all gene expressions in each module (Figure 3B). Meanwhile, the eigengene of all modules was calculated and clustered based on the correlation via Pearson correlation coefficients between pairwise (Figure 3C). Further, the relevance of eigengenes of each module and taxanes-sensitivity were assessed respectively *via* Module-Trait Relationships (MTRs). The results showed eigengenes of the yellow module presented the highest positive correlation with paclitaxel therapeutic effectiveness (Figure 3D). The results in Figure 3E showed an evidently positive correlation (*r* = 0.51, *p* = 0.0002) between gene significance (GS) for taxanes-sensitivity and module membership (MM) in the yellow module. Therefore, genes in the yellow module were selected for subsequent analysis. To further identify hub genes regulating the biological characteristics in the yellow module, the weight coefficient was calculated between the pairwise gene. Most genes relevant to cytokine-cytokine receptor interaction showed a larger weight coefficient (Figure 3F).

### Hub Genes Were Identified to Be Associated With Taxanes-Resistance and Prognosis of Breast Cancers

**Figure 4:**
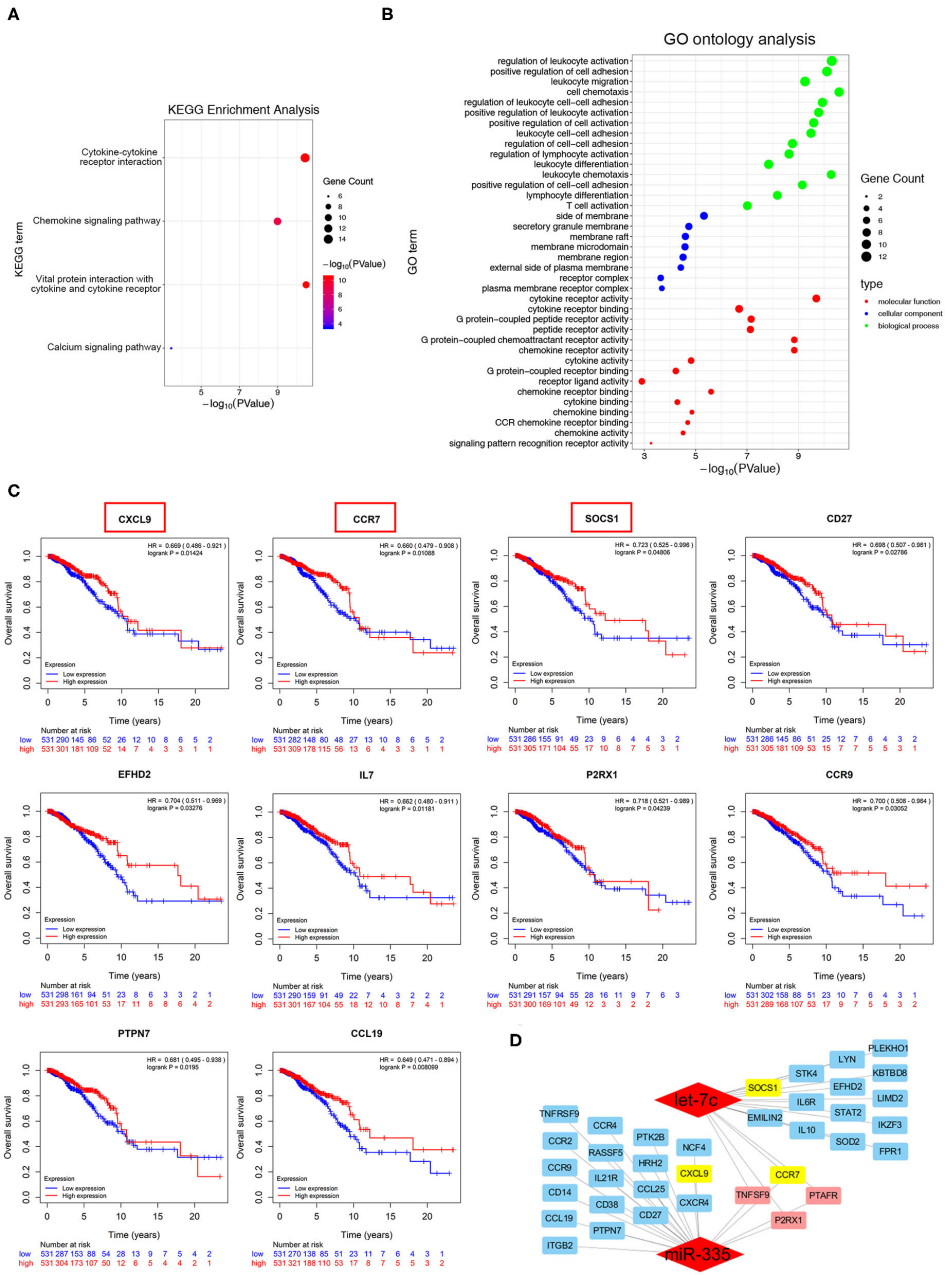
Identification of potential biological roles of the hub genes in the yellow module. **(A,B)** KEGG pathway enrichment and GO ontology analysis of all genes in the yellow module. **(C)** Overall survival curves based on hub genes expression of breast cancer patients in TCGA database. **(D)** Sub-network of candidate target genes and the corresponding upstream miRNAs.

The potential target genes in the yellow module were submitted in KEGG pathway enrichment and GO ontology analysis. As shown in Figure 4A, most genes were associated with cytokine-cytokine receptor interactions, chemokine signaling pathways, and Jak-STAT signaling pathways. The following GO ontology analysis showed genes enriched in the regulation of phospholipid metabolic process, lipid kinase activity, immune cells costimulation, and chemotaxis (Figure 4B). Furthermore, survival analysis showed among candidate target genes, CXCL9, CCR7, SOCS1, CD27, EFHD2, IL7, P2RX1, CCR9, PTPN7, and CCL19 were expressed as a better prognosis of breast cancer patients in the TCGA database (*n* = 1062) (Figure 4C). To the summary, the network in Figure 4D depicted the candidate hub genes relevant to taxanes-sensitivity respectively in the downstream of miR-335-5p and let-7c-5p.

### CXCL9, CCR7, and SOCS1 are Identified as Target Genes of miR-335-5p and Let-7c-5p

**Figure 5:**
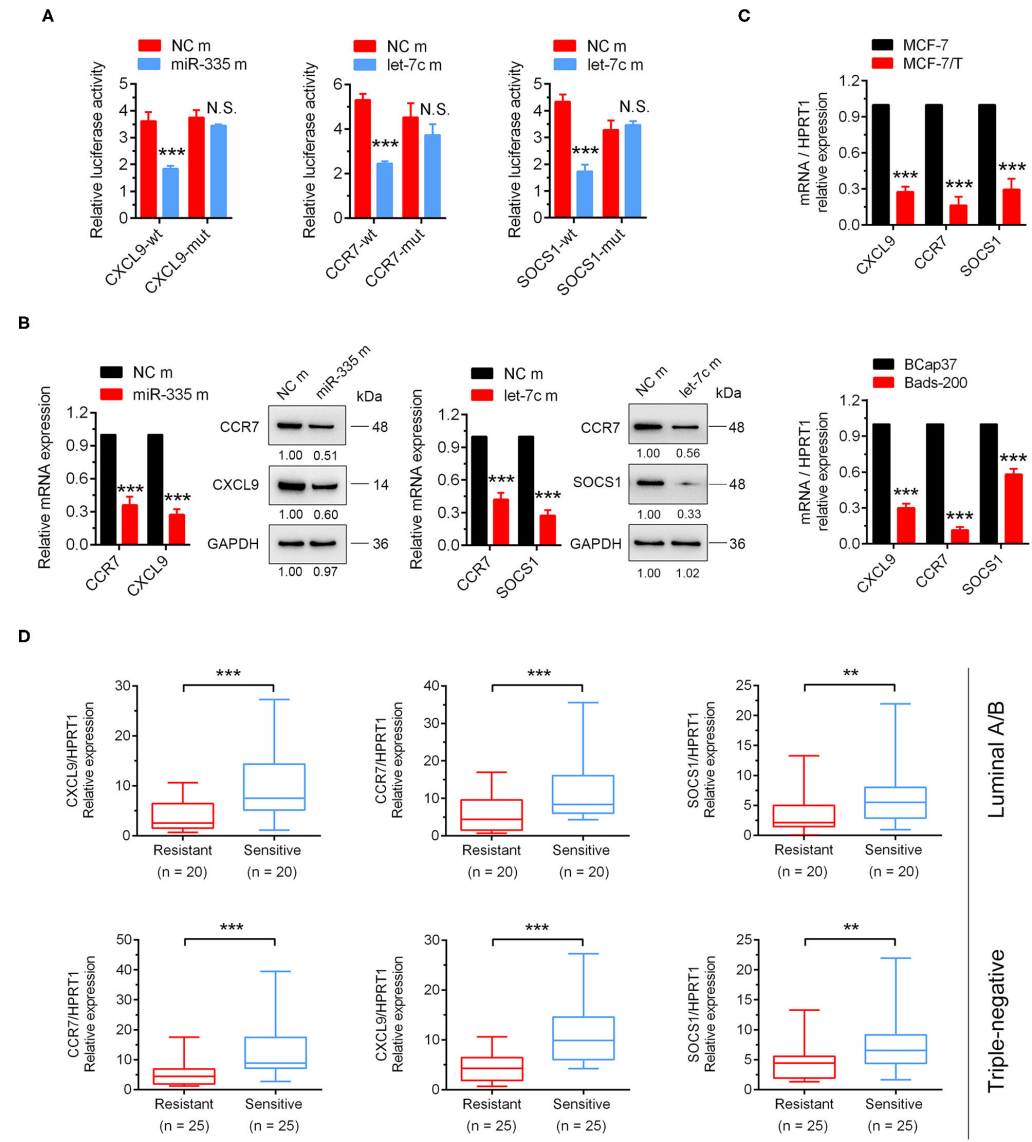
CXCL9, CCR7/SOCS1 were validated as the direct target genes of miR-335-5p and let-7c-5p, respectively. **(A)** The dual-luciferase assays indicated miR-335-5p could directly target CXCL9, and let-7c-5p could directly bind to CCR7 and SOCS1. **(B)** The results of qRT-PCR and Western Blot indicated CXCL9 and CCR7 could be inhibited by miR-335-5p, CCR7, and SOCS1 could be inhibited by let-7c-5p both in mRNA and protein levels. **(C)** These three target genes were downregulated in paclitaxel-resistant cell lines (MCF-7/T and Bads-200). **(D)** CXCL9, CCR7, and SOCS1 were downregulated tumor samples of taxanes-resistant group in clinic. ^**^P < 0.01, ^***^P < 0.001.

To further identify target genes in the downstream of miR-335-5p and let-7c-5p, the dual-luciferase assay was performed. The results showed that miR-335-5p overexpression could notably decrease the luciferase activities of the wild-type 3'UTR reporters of CXCL9, together with let-7c-5p could significantly inhibit the luciferase activities of CCR7 and SOCS1. Further, the mutant binding sites were constructed into the luciferase reporter isotopic, and the results showed neither miR-335-5p nor let-7c-5p could attenuate the luciferase activities (Figure 5A). The outcomes strongly indicated CXCL9 was the direct target of miR-335-5p, and CCR7, and SOCS1 were direct targets of let-7c-5p. Intriguingly, the mRNA and protein expression of CXCL9 and CCR7 were both significantly inhibited by ectopic overexpression of miR-335-5p, in the meanwhile, CCR7 and SOCS1 were attenuated by let-7c-5p (Figure 5B, Figure S2D). However, the outcomes of dual-luciferase reporter assay didn't show the direct binding of miR-335-5p and CCR7. We suggested another potential regulatory mechanism might exist, which would be explored in subsequent research. More importantly, the mRNA expressions of CXCL9, CCR7, and SOCS1 in breast cancer cells were observed notably higher in the taxanes-sensitive cell lines (MCF-7 and BCap37) than taxanes-resistant cell lines (MCF-7/T and Bads-200) (Figure 5C). Such similar results were observed in breast cancer tissues both of luminal and triple-negative subtypes (Figure 5D). In total, the results strongly suggested CXCL9, CCR7, and SOCS1 played an important role in chemo-resistance of breast cancer.

### Upregulation of CXCL9, CCR7, and SOCS1 Suppressed Cell Proliferation and Taxanes-Resistance, Promoted Apoptosis in Breast Cancer Cells

**Figure 6:**
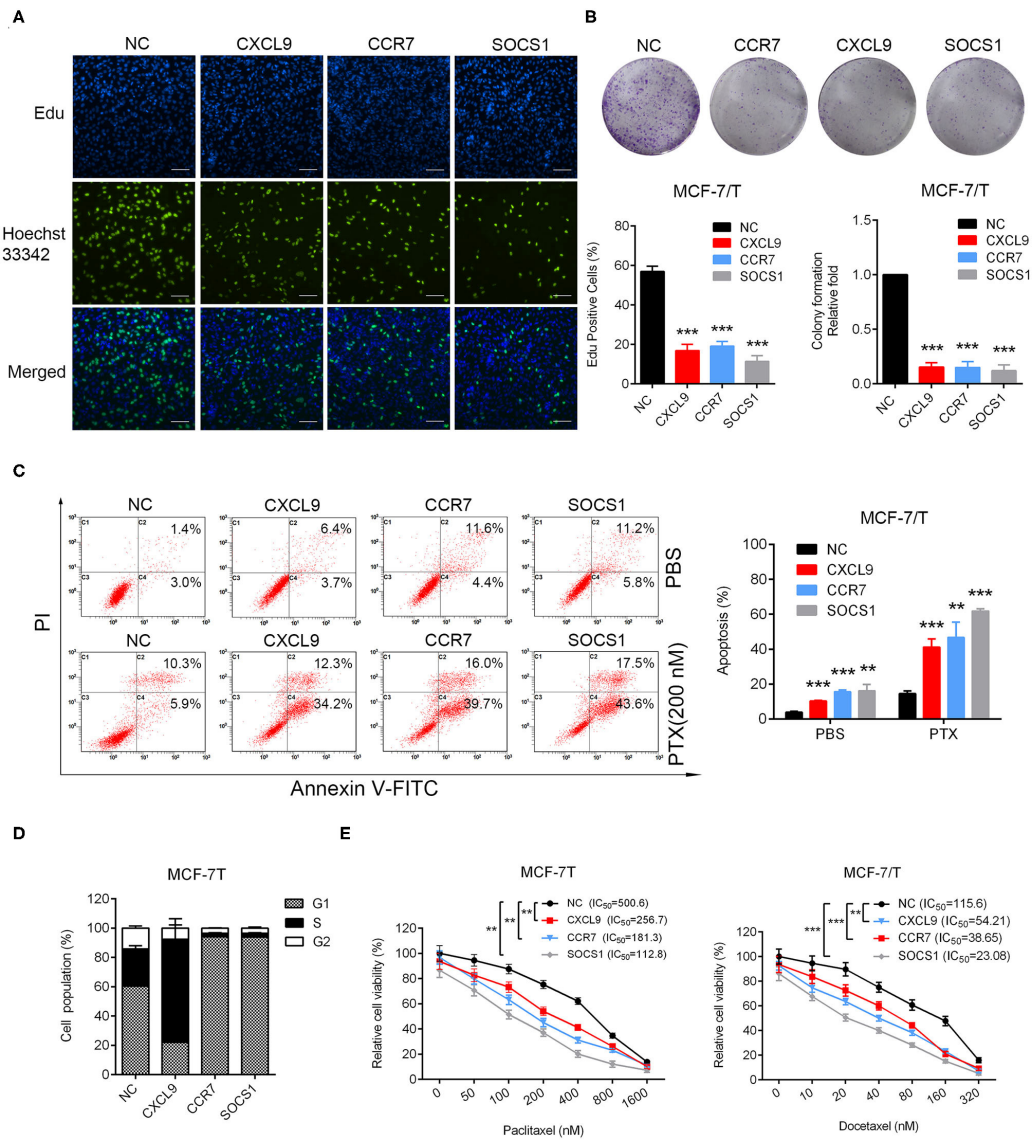
Identification of biological functions of CXCL9, CCR7, and SOCS1. **(A)** Edu incorporation assays, **(B)** colony formation assays, **(C)** Apoptosis rate was detected in CXCL9/CCR7/SOCS1-overexpressing MCF-7/T cells. **(D)** Cell cycle assays were performed in CXCL9/CCR7/SOCS1-overexpressing MCF-7/T cells. **(E)** The IC_50_ to paclitaxel and docetaxel was detected by MTT assays. All experiments were performed at least three times independently. The data were presented as the mean ± *SD*. ^**^P < 0.01, ^***^P < 0.001. Scale bar: 500 μM.

In order to confirm the biological functions of CXCL9, CCR7, and SOCS1 in breast cancer, the plasmids for overexpressing CXCL9, CCR7, and SOCS1 were transfected into MCF-7/T cells (Figure S2E). Results of Edu and colony formation assays showed cell proliferation and colonogenicity were notably inhibited (Figures 6A,B). The apoptosis levels were elevated with or without combinational effect of paclitaxel (Figure 6C). More cells were arrested in G1 stage by overexpression of CCR7 and SOCS1, and more cells arrested in S stage by upregulation of CXCL9 (Figure 6D). Further, the IC_50_ of paclitaxel and docetaxel were significantly reduced (Figure 6E). All the outcomes strongly confirmed that CXCL9, CCR7, and SOCS1 screened out *via* WGCNA participated in the regulation of taxanes-sensitivity in breast cancer cells. The outcomes strongly suggested a ubiquitous mechanism of CXCL9, CCR7, and SOCS1 in chemo-resistance, except a conventional function in immune system.

## Discussion

Chemo-resistance to multitudinous anticancer agents emerges and severely limits therapeutic efficacy in clinic. Over the past decade, a large number of researches have focused on exploring the role of miRNA-mRNA axes played in chemo-resistance. Plenty of miRNA-mRNA axes, such as miR-34a-Notch1 axis (30), miR-125b-Sema4c axis (31), miR-181-Bcl-2 axis (33), and miR-27b/CBLB/GRB2 axis (34), were reported that regulated taxanes-resistance in breast cancer. However, the functions of several miRNAs were controversial, even the contrary in different tumor context or cell lines. In our opinion, this discrepancy may be attributed by different miRNA-mRNA interactional networks for different instances. As is known, a single miRNA can regulate multiple target genes and affect various signaling pathways in diverse cellular activities. Simultaneously the expression of the miRNA could be regulated by amounts of regulatory factors, including genes, other non-coding RNAs. Therefore, the intricate networks should be considered to understand the integral functions of miRNA-mRNA axes (35).

**Figure 7:**
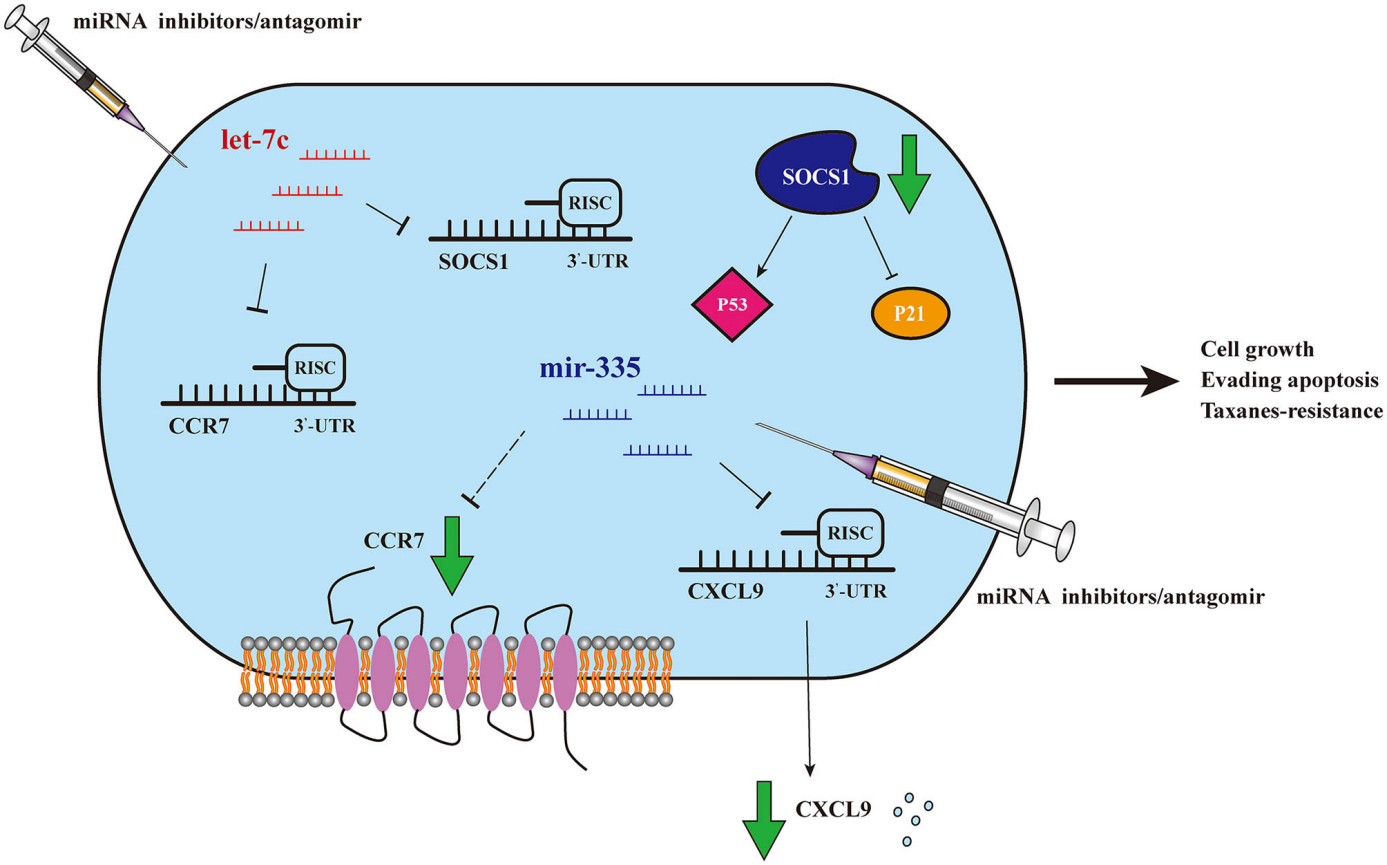
Schematic model of miR-335-5p/CXCL9, let-7c-5p/CCR7/SOCS1 axes. Schematic model of miR-335-5p and let-7c-5p may regulate proliferation, apoptosis, and taxanes-resistance in breast cancer, by mediating CXCL9, CCR7, and SOCS1.

In the present study, 12 dysregulated miRNAs were screened out by exhaustive analysis of thousands of miRNAs expressions in breast cancer samples between taxanes-resistant and taxanes-sensitive groups in TCGA database. Further, upregulated expression of three miRNAs (miR-335-5p, let-7c-5p, and miR-99a-5p) were validated in clinical taxanes-resistant breast cancer samples and paclitaxel-resistant cell lines. The consistent trends strongly confirmed our preliminary screening results. Importantly, we confirmed that dysregulation of miR-335-5p and let-7c-5p could notably effect cells proliferation, apoptosis level and IC_50_ of paclitaxel and docetaxel. However, similar outcomes were not observed by knockdown of miR-99a-5p. We speculated the upregulated expression of miR-99a-5p in taxanes-resistant context was a satellite phenomenon, and it may not play a leading role. Therefore, miR-99a-5p was not the focus in this study. After that, we emphasized accurately screening out target genes involved in taxanes-resistance in the downstream of miR-335-5p and let-7c-5p. WGCNA analysis was conducted and figured out a group of potential target genes with positive relationship to taxanes-sensitivity. Survival analysis showed that high expressions of several genes were strongly associated with good prognosis. Finally, we confirmed that miR-335-5p could inhibit expression of CXCL9, and let-7c-5p could suppress expressions of CCR7 and SOCS1, both by directly binding to the 3'UTR of the genes. Intriguingly, miR-335-5p was observed that inhibited expression of CCR7 both in mRNA and protein level, without directly binding. We speculated other mechanisms might be involved in the regulation, which deserved further research. The Figure 7 schematically presented the potential mechanisms of miR-335-5p/CXCL9, let-7c-5p/CCR7/SOCS1 axes involved in regulating proliferation, apoptosis, and taxanes-resistance in breast cancer.

miR-335 is commonly recognized as a tumor suppressor mainly mediating tumorigenesis and metastasis. In gastric cancer, exogenous miR-335 decreased migration and invasion of AGS and Hs 746T cells by inhibiting PLAUR and CDH11 genes (36). In hepatocellular carcinoma (HCC), miR-335 loaded in extracellular vesicles, which were taken up by HCC cells, could inhibit cell proliferation and invasion (37). In ovarian cancer, upregulation of miR-335 could enhance cisplatin-induced apoptosis through suppressing BCL2L2 (38). Like most miRNAs, controversial results of miR-335 remain in a cell context-dependent manner. In breast cancer, upregulation of miR-335-5p/-3p was reported to enhance tamoxifen-resistance of MCF-7 cells, by influencing ERα signaling pathway (39). Let-7c was reported to function as tumor suppressor in prostate cancer by regulating AR transcription via MYC (40). In mucosal melanoma, the expressions of let-7c and let-7b were decreased, whereas overexpressing of them could increase sensitivity to temozolomide and paclitaxel by inhibiting MTDH (41). In addition, ectopic expression of let-7c could suppressed migration and invasion ability of cholangiocarcinoma cells *in vitro*, while increase distant invasiveness *in vivo*. The difference partly depended on dual role of EZH2 and AVL3/β-catenin axis (42). In our study, we indicated that both of miR-335-5p and let-7c-5p exhibited “oncogene” role to induce taxanes-resistance in breast cancer. We speculate the contradictions mainly depend on target genes in the downstream of miRNAs.

Chemokines were reported as key factors in immunological homeostasis (43), but also acted as activators and/or inhibitors in oncogenesis, by influencing tumor growth, epithelial-mesenchymal transition (EMT), metastasis (44), cell-cycle progression (45), and angiogenesis (46, 47). CXCL9, one of CXC chemokine family, plays a dramatic role in the chemotaxis of immune cells. CXCL9 is secreted by various types of cells, including immune cells (e.g., macrophages, NK cells, dendritic cells, T lymphocytes), and non-immune cells (e.g., tumor cells and endothelial cells) (48). The main mechanism of CXCL9 in regulating immune activity is to facilitate chemotactic recruitment of tumor infiltrating lymphocytes (TILs) (49, 50). In high-grade ovarian cancer, a study reported intratumoral accumulation of CXCL9 could act as tumor suppressor by significantly improving TIL-dependent immune intervention (51). In addition, CXCL9,−10,−11/CXCR3 network was confirmed to affect tumor resistance to checkpoint inhibitors by regulating immune cells activation (52). Moreover, upregulation of CXCL9 was observed in combinational therapy of anti-TIM-3 and paclitaxel, and which was validated to reverse paclitaxel-resistance (53).

C-C chemokine receptor type 7 (CCR7), is one of members of the chemokine receptor superfamily and its ligand is CCL19. It has been recognized that CXCL9/CCR7 axis mediated various cellular behaviors, mainly by influencing immune response evasion (54). In tumorigenesis, most studies emphasized on the relationship of CXCL9/CCR7 with tumor angiogenesis and metastasis (55, 56). In NSCLC, a study observed an upregulation of CXCL9/CCR7 in tumor tissue, especially in patients with metastasis to lymph nodes. Intriguingly, the expression of CXCL9/CCR7 were associated with two miRNAs: let-7a and miR-335 (57). In addition, high expression of CXCL9 was observed in melanoma-derived cancer stem cells (CSC), which may accelerate efficient recognition and kill by NK cells (58). SOCS1 was recognized as a potent inhibitor of inflammation in immune cells by negatively regulating cytokine-activated JAK-STAT pathway (59). Recently SOCS1 was viewed as a tumor suppressor in types of tumors, and the mechanisms mainly include activating P53 tumor suppressor functions (60–62), inhibiting P21 oncogenic functions (63, 64), and RTK signaling pathways (62). Up to now, few researches indicated that CCR7, SOCS1 were related with chemo-resistance, and we would venture to assume that miR-335-5p/CXCL9, let-7c-5p/CCR7/SOCS1 may play a part in regulating taxanes-resistance by mediating interaction of immune cells. In subsequent study, we would like to explore the further mechanisms to figure it out.

In conclusion, we introduced miR-335-5p and let-7c-5p as potent regulatory factors resulting in taxanes-resistance in breast cancer cells. Further, target genes (CXCL9, CCR7, and SOCS1) were confirmed in the downstream of these two miRNAs. Both of miR-335-5p/CXCL9 and let-7c-5p/CCR7/SOCS1 axes were validated with relationship to taxanes-resistant phenotypes. To the best of our knowledge, it's the first time linking the regulation of cytokine networks by miRNAs and its role in chemo-resistance. Moreover, miR-335-5p/CXCL9 and let-7c-5p/CCR7/SOCS1 axes may be involved in mechanism of immune cells participating in chemo-resistance, which needs further exploration.

## Data Availability Statement

Publicly available datasets were analyzed in this study, these can be found in The Cancer Genome Atlas (TCGA) data portal (https://genome-cancer.ucsc.edu/). Relevant datasets generated for this study are included in the article/Supplementary Material.

## Ethics Statement

The studies involving human participants were reviewed and approved by Clinical Research Ethics Committee of the First Affiliated Hospital, College of Medicine, Zhejiang University. The patients/participants provided their written informed consent to participate in this study.

## Author Contributions

DC, CB, and FZ: conceived the study and analyzed data. DC and CB: designed and performed the experiments. DC: wrote the manuscript. SY and FZ: revised the manuscript. GZ, HY, and LX: contributed reagents/clinical samples/analysis tools. All authors read and approved the final manuscript.

## Conflict of Interest

The authors declare that the research was conducted in the absence of any commercial or financial relationships that could be construed as a potential conflict of interest.
