# Supplementary material for: Exploring Specific miRNA-mRNA Axes With Relationship to Taxanes-Resistance in Breast Cancer
Source: Front Oncol. 2020 Aug 21;10:1397. doi: 10.3389/fonc.2020.01397 (PMC7473300; doi:10.3389/fonc.2020.01397)
Supplement: Supplementary file 1 [file Table_1.docx]

Table S1 Sensitivity to chemotherapeutic drugs of MCF-7 and MCF-7/T cells

| **Drugs** | **MCF-7** | **MCF-7/T** | **RI** |
| --- | --- | --- | --- |
|  | **IC_50_ (nM)** | **IC_50_ (nM)** |  |
| Paclitaxel (PTX) | 18.33 ± 4.16 | 513.7 ± 27.7’ | **28.02** |
| Docetaxel (DOC) | 8.22 ± 0.99 | 121.7 ± 17.6 | **14.81** |
| Doxorubicin (DOX) | 111.7 ± 15.0 | 966.3 ± 54.3 | 8.65 |
| Gemcitabin (GEM) | 1494 ± 316.1 | 7494 ± 316.1 | 5.02 |
| Cisplatin (DDP) | 64.67 ± 10.70 | 1171 ± 42.71 | **18.11** |

IC_50_ means the drug concentration could inhibit 50% cells proliferation.

RI (Drug resistance index) means IC_50_ of resistant-cell line/ IC_50_ of sensitive-cell line.
