# Supplementary material for: Exploring Specific miRNA-mRNA Axes With Relationship to Taxanes-Resistance in Breast Cancer
Source: Front Oncol. 2020 Aug 21;10:1397. doi: 10.3389/fonc.2020.01397 (PMC7473300; doi:10.3389/fonc.2020.01397)
Supplement: Supplementary file 2 [file Table_2.docx]

Table S2 Information of selected miRNAs applied in the present study

| **Name** | **miRbase** | **mature sequence (5’-3’)** | |
| --- | --- | --- | --- |
| hsa-let-7c-5p | MIMAT0000064 | Mimic | ugagguaguagguuguaugguu  acuccaucauccaacauaccaa |
|  |  | Inhibitor | acuccaucauccaacauaccaa |
| hsa-mir-335-5p | MIMAT0000765 | Mimic | ucaagagcaauaacgaaaaaugu  aguucucguuauugcuuuuaca |
|  |  | Inhibitor | aguucucguuauugcuuuuaca |
| hsa-mir-99a-5p | MIMAT0000097 | Mimic | aacccguagauccgaucuugug  uugggcaucuaggcuagaacac |
|  |  | Inhibitor | uugggcaucuaggcuagaacac |
