# Supplementary material for: Exploring Specific miRNA-mRNA Axes With Relationship to Taxanes-Resistance in Breast Cancer
Source: Front Oncol. 2020 Aug 21;10:1397. doi: 10.3389/fonc.2020.01397 (PMC7473300; doi:10.3389/fonc.2020.01397)
Supplement: Supplementary file 3 [file Table_3.docx]

Table S3 Information of primers applied in the present study

| **Primers** | **5’-3’** |
| --- | --- |
| qRT-PCR |  |
| CXCL9-F | TCTTGCTGGTTCTGATTGGAGTGC |
| CXCL9-R | GTCCCTTGGTTGGTGCTGATGC |
| CCR7-F | GTCCCTTGGTTGGTGCTGATGC |
| CCR7-R | GGGTGCGGATGATGACAAGGTAAC |
| SOCS1-F | CCAGGTGGCAGCCGACAATG |
| SOCS1-R | CGAGGAGGAGGAAGAGGAGGAAG |
| Wild-3’UTR |  |
| CXCL9-F | aattctaggcgatcgctcgagAGCAAATAATTTTTCACTTCAAAACAG |
| CXCL9-R | attttattgcggccagcggccgcGTGTTGTCATATTTTCCATAGGTTTTT |
| CCR7-F | aattctaggcgatcgctcgagGCGACTCTTCTGCCTGGACTAG |
| CCR7-R | attttattgcggccagcggccgcCAGGTGGGAACAGTTTCTGGAC |
| SOCS1-F | aattctaggcgatcgctcgagTGGGTGTAGGGGCGAGGC |
| SOCS1-R | attttattgcggccagcggccgcTAATAAAGTTTATTACCTAAACTGAC |
| Mut-3’UTR |  |
| CXCL9-F | TTgagaactATGTACCCTTCAACTGTTGAATGTTT |
| CXCL9-R | GGGTACATagttctcAAAAAAAATCAAGATCTGTTTTCTGATT |
| CCR7-F | TTgagaactATGTACCCTTCAACTGTTGAATGTTT |
| CCR7-R | GGGTACATagttctcAAAAAAAATCAAGATCTGTTTTCTGATT |
| SOCS1-F | ACCTCgatggagTTCATGTTTACATATACCCAGTATCTTTG |
| SOCS1-R | ATGAActccatcGAGGTGCGAGTTCAGGTCCTG |
