# Supplementary material for: Exploring Specific miRNA-mRNA Axes With Relationship to Taxanes-Resistance in Breast Cancer
Source: Front Oncol. 2020 Aug 21;10:1397. doi: 10.3389/fonc.2020.01397 (PMC7473300; doi:10.3389/fonc.2020.01397)
Supplement: Supplementary file 4 [file Table_4.docx]

Table S4 Information of breast cancer patients in clinic

| **No** | **Age** | **Subtypes** | [**Histological**](javascript:;) [**Grade**](javascript:;) | | **Chemotherapy** | | **Response** |
| --- | --- | --- | --- | --- | --- | --- | --- |
| 1 | 55 | Luminal A | II | paclitaxel | | CR | |
| 2 | 46 | Luminal A | III | paclitaxel | | CR | |
| 3 | 48 | Luminal A | II-III | paclitaxel | | CR | |
| 4 | 46 | Luminal A | II | paclitaxel | | CR | |
| 5 | 39 | Luminal A | II-III | paclitaxel | | CR | |
| 6 | 61 | Luminal A | III | paclitaxel | | CR | |
| 7 | 39 | Luminal A | III | paclitaxel | | CR | |
| 8 | 57 | Luminal A | II | paclitaxel | | CR | |
| 9 | 41 | Luminal A | III | paclitaxel | | CR | |
| 10 | 67 | Luminal A | II | paclitaxel | | CR | |
| 11 | 43 | Luminal A | III | paclitaxel | | CR | |
| 12 | 49 | Luminal A | III | paclitaxel | | CR | |
| 13 | 59 | Luminal B | II | paclitaxel | | CR | |
| 14 | 62 | Luminal B | III | paclitaxel | | PR | |
| 15 | 68 | Luminal B | II-III | paclitaxel | | PR | |
| 16 | 55 | Luminal B | II | paclitaxel | | CR | |
| 17 | 65 | Luminal B | III | paclitaxel | | PR | |
| 18 | 64 | Luminal B | II-III | paclitaxel | | PR | |
| 19 | 59 | Luminal B | II | paclitaxel | | CR | |
| 20 | 69 | Luminal B | III | paclitaxel | | PR | |
| 21 | 63 | Luminal A | II-III | paclitaxel | | SD | |
| 22 | 53 | Luminal A | II | paclitaxel | | SD | |
| 23 | 48 | Luminal A | III | paclitaxel | | SD | |
| 24 | 43 | Luminal A | III | paclitaxel | | SD | |
| 25 | 46 | Luminal A | III | paclitaxel | | SD | |
| 26  626 | 57 | Luminal A | II-III | paclitaxel | | SD | |
| 27 | 51 | Luminal A | II | paclitaxel | | SD | |
| 28 | 49 | Luminal A | III | paclitaxel | | SD | |
| 29 | 46 | Luminal A | III | paclitaxel | | SD | |
| 30 | 31 | Luminal A | III | paclitaxel | | SD | |
| 31 | 41 | Luminal B | III | paclitaxel | | SD | |
| 32 | 63 | Luminal B | I | paclitaxel | | SD | |
| 33 | 48 | Luminal B | III | paclitaxel | | SD | |
| 34 | 38 | Luminal B | II | paclitaxel | | SD | |
| 35 | 44 | Luminal B | I | paclitaxel | | SD | |
| 36 | 41 | Luminal B | III | paclitaxel | | PD | |
| 37 | 50 | Luminal B | III | paclitaxel | | PD | |
| 38 | 45 | Luminal B | III | paclitaxel | | PD | |

Table S4 Information of breast cancer patients in clinic

| **No** | **Age** | **Subtypes** | [**Histological**](javascript:;) [**Grade**](javascript:;) | | **Chemotherapy** | **Response** | |
| --- | --- | --- | --- | --- | --- | --- | --- |
| 39 | 35 | Luminal B | III | paclitaxel | | | PD |
| 40 | 57 | Luminal B | III | paclitaxel | | | PD |
| 41 | 39 | TNBC | II | paclitaxel | | | CR |
| 42 | 63 | TNBC | II | paclitaxel | | | CR |
| 43 | 67 | TNBC | II | paclitaxel | | | CR |
| 44 | 72 | TNBC | II | paclitaxel | | | CR |
| 45 | 55 | TNBC | III | paclitaxel | | | CR |
| 46 | 58 | TNBC | II | paclitaxel | | | CR |
| 47 | 57 | TNBC | II | paclitaxel | | | CR |
| 48 | 52 | TNBC | I | paclitaxel | | | CR |
| 49 | 65 | TNBC | II | paclitaxel | | | CR |
| 50 | 59 | TNBC | I | paclitaxel | | | CR |
| 51 | 60 | TNBC | II | paclitaxel | | | CR |
| 52 | 52 | TNBC | III | paclitaxel | | | CR |
| 53 | 69 | TNBC | III | paclitaxel | | | CR |
| 54 | 76 | TNBC | I-II | paclitaxel | | | CR |
| 55 | 65 | TNBC | III | paclitaxel | | | CR |
| 56 | 47 | TNBC | II | paclitaxel | | | CR |
| 57 | 56 | TNBC | I-II | paclitaxel | | | CR |
| 58 | 62 | TNBC | II-III | paclitaxel | | | CR |
| 59 | 42 | TNBC | II-III | paclitaxel | | | CR |
| 60 | 58 | TNBC | III | paclitaxel | | | CR |
| 61 | 59 | TNBC | II | paclitaxel | | | PR |
| 62 | 47 | TNBC | II | paclitaxel | | | PR |
| 63 | 65 | TNBC | II | paclitaxel | | | PR |
| 64 | 68 | TNBC | II | paclitaxel | | | PR |
| 65 | 69 | TNBC | III | paclitaxel | | | PR |
| 66 | 68 | TNBC | II | paclitaxel | | | SD |
| 67 | 57 | TNBC | III | paclitaxel | | | SD |
| 68 | 57 | TNBC | II | paclitaxel | | | SD |
| 69 | 54 | TNBC | II | paclitaxel | | | SD |
| 70 | 48 | TNBC | III | paclitaxel | | | SD |
| 71 | 47 | TNBC | II | paclitaxel | | | SD |
| 72 | 80 | TNBC | II | paclitaxel | | | SD |
| 73 | 81 | TNBC | II | paclitaxel | | | SD |
| 74 | 67 | TNBC | III | paclitaxel | | | SD |
| 75 | 61 | TNBC | III | paclitaxel | | | SD |
| 76 | 59 | TNBC | III | paclitaxel | | | SD |

Table S4 Information of breast cancer patients in clinic

| **No** | **Age** | **Subtypes** | [**Histological**](javascript:;) [**Grade**](javascript:;) | | **Chemotherapy** | **Response** | |
| --- | --- | --- | --- | --- | --- | --- | --- |
| 77 | 32 | Luminal B | II | paclitaxel | | | SD |
| 78 | 42 | Luminal B | II | paclitaxel | | | SD |
| 79 | 37 | TNBC | II | paclitaxel | | | SD |
| 80 | 45 | TNBC | II | paclitaxel | | | \| SD \| \| --- \| \| SD \| \| SD \|   YES |
| 81 | 40 | TNBC | II | paclitaxel | | | \| SD \| \| --- \| \| SD \| \| SD \|   YES |
| 82 | 57 | TNBC | II | paclitaxel | | | \| SD \| \| --- \| \| SD \| \| SD \|   YES |
| 83 | 57 | TNBC | III | paclitaxel | | | PD |
| 84 | 51 | TNBC | II | paclitaxel | | | PD |
| 85 | 59 | TNBC | II | paclitaxel | | | PD |
| 86 | 52 | TNBC | I | paclitaxel | | | PD |
| 87 | 63 | TNBC | II | paclitaxel | | | PD |
| 88 | 58 | TNBC | I | paclitaxel | | | PD |
| 89 | 60 | TNBC | II | paclitaxel | | | PD |
| 90 | 62 | TNBC | II | paclitaxel | | | PD |

All information was collected in Breast Center, the First Affiliated Hospital, College of Medicine, Zhejiang University. This study was approved by the Research Ethics Committee of the First Affiliated Hospital, College of Medicine, Zhejiang University.

CR, Complete Response.

PR, Partial Response.

SD, Stable Disease.

PD, Progressive Disease.
