# Supplementary material for: Exploring Specific miRNA-mRNA Axes With Relationship to Taxanes-Resistance in Breast Cancer
Source: Front Oncol. 2020 Aug 21;10:1397. doi: 10.3389/fonc.2020.01397 (PMC7473300; doi:10.3389/fonc.2020.01397)
Supplement: Supplementary file 5 [file Table_5.docx]

Table S5 Information of breast cancer patients in TCGA database

| **No** | **Id** | **Age** | **Time(d)** | | **Status** | **TNM** | **Chemotherapy** | **Response** | |
| --- | --- | --- | --- | --- | --- | --- | --- | --- | --- |
| 1 | TCGA-A2-A0EP | 56 | 3273 | Alive | | I | Paclitaxel | CR |  |
| 2 | TCGA-BH-A0W7 | 49 | 1363 | Alive | | I | Taxotere | CR |  |
| 3 | TCGA-BH-A18H | 63 | 652 | Alive | | I | Taxotere | CR |  |
| 4 | TCGA-E2-A573 | 48 | 1062 | Alive | | I | Taxotere | CR |  |
| 5 | TCGA-GM-A2DH | 58 | 2193 | Alive | | I | Taxol | CR |  |
| 6 | TCGA-GM-A2DI | 52 | 2590 | Alive | | I | Taxotere | CR |  |
| 7 | TCGA-LL-A440 | 61 | 759 | Alive | | I | Taxotere | CR |  |
| 8 | TCGA-LL-A441 | 62 | 996 | Alive | | I | Taxotere | CR |  |
| 9 | TCGA-LL-A5YO | 50 | 440 | Alive | | I | Taxotere | CR |  |
| 10 | TCGA-LL-A740 | 61 | 441 | Alive | | I | Taxol | CR |  |
| 11 | TCGA-OL-A6VO | 43 | 858 | Alive | | I | Taxotere | CR |  |
| 12 | TCGA-S3-AA14 | 47 | 455 | Alive | | I | Taxol | CR |  |
| 13 | TCGA-Z7-A8R6 | 46 | 3256 | Alive | | I | Taxol | CR |  |
| 14 | TCGA-B6-A402 | 47 | 2281 | Alive | | I | Paclitaxel | CR |  |
| 15 | TCGA-A2-A3XV | 46 | 699 | Alive | | II | Docetaxel | CR |  |
| 16 | TCGA-A2-A3XW | 42 | 1505 | Alive | | II | Taxol | CR |  |
| 17 | TCGA-A2-A3XX | 49 | 1439 | Death | | II | Paclitaxel | CR |  |
| 18 | TCGA-A2-A3Y0 | 57 | 1167 | Alive | | II | Docetaxel | CR |  |
| 19 | TCGA-A7-A4SA | 40 | 454 | Alive | | II | Paclitaxel | CR |  |
| 20 | TCGA-A7-A4SD | 52 | 441 | Alive | | II | Taxotere | CR |  |
| 21 | TCGA-A7-A4SE | 54 | 644 | Alive | | II | Taxol | CR |  |
| 22 | TCGA-A7-A4SF | 54 | 545 | Alive | | II | Docetaxel | CR |  |
| 23 | TCGA-A7-A5ZV | 62 | 368 | Alive | | II | Paclitaxel | CR |  |
| 24 | TCGA-A7-A5ZW | 47 | 326 | Alive | | II | Taxotere | CR |  |
| 25 | TCGA-A7-A6VV | 51 | 313 | Alive | | II | Taxotere | CR |  |
| 26  626 | TCGA-A7-A6VW | 48 | 285 | Alive | | II | Taxol | CR |  |
| 27 | TCGA-A7-A6VX | 68 | 317 | Alive | | II | Paclitaxel | CR |  |
| 28 | TCGA-A7-A6VY | 48 | 266 | Alive | | II | Paclitaxel | CR |  |
| 29 | TCGA-A8-A075 | 42 | 518 | Alive | | II | Docetaxel | CR |  |
| 30 | TCGA-A8-A07C | 57 | 1034 | Alive | | II | Docetaxel | CR |  |
| 31 | TCGA-A8-A07J | 35 | 365 | Alive | | II | Docetaxel | CR |  |
| 32 | TCGA-A8-A082 | 58 | None | None | | II | Docetaxel | CR |  |
| 33 | TCGA-A8-A086 | 59 | 396 | Alive | | II | Docetaxel | CR |  |
| 34 | TCGA-A8-A08G | 41 | 607 | Alive | | II | Docetaxel | CR |  |
| 35 | TCGA-A8-A097 | 65 | 365 | Alive | | II | Docetaxel | CR |  |
| 36 | TCGA-A8-A09D | 47 | 1522 | Alive | | II | Paclitaxel | CR |  |
| 37 | TCGA-AC-A23G | 50 | 2248 | Alive | | II | Taxol | CR |  |
| 38 | TCGA-AC-A2FB | 65 | 1234 | Alive | | II | Taxotere | CR |  |

Table S5 Information of breast cancer patients in TCGA database

| **No** | **Id** | **Age** | **Time(d)** | **Status** | **TNM** | **Chemotherapy** | | **Response** |
| --- | --- | --- | --- | --- | --- | --- | --- | --- |
| 39 | TCGA-AC-A2FF | 40 | 2420 | Alive | II | Taxol | CR | |
| 40 | TCGA-AC-A3W5 | 65 | 504 | Alive | II | Docetaxel | CR | |
| 41 | TCGA-BH-A0B7 | 42 | 2559 | Alive | II | Taxol | CR | |
| 42 | TCGA-BH-A0C7 | 48 | 2767 | Alive | II | Taxol | CR | |
| 43 | TCGA-BH-A0DG | 30 | 2041 | Alive | II | Taxotere | CR | |
| 44 | TCGA-BH-A0DI | 63 | 912 | Alive | II | Taxol | CR | |
| 45 | TCGA-BH-A0DL | 64 | 2381 | Alive | II | Taxotere | CR | |
| 46  7 | TCGA-BH-A0DT | 41 | 2403 | Alive | II | Taxotere | CR | |
| 47 | TCGA-BH-A0E9 | 53 | 2489 | Alive | II | Taxotere | CR | |
| 48 | TCGA-BH-A0EA | 62 | 991 | Death | II | Taxotere | CR | |
| 49 | TCGA-BH-A18F | 50 | 1001 | Alive | II | Taxotere | CR | |
| 50 | TCGA-BH-A18I | 53 | 1093 | Alive | II | Taxotere | CR | |
| 51 | TCGA-BH-A202 | 60 | 795 | Alive | II | Taxotere | CR | |
| 52 | TCGA-BH-A5IZ | 51 | 567 | Alive | II | Taxotere | CR | |
| 53 | TCGA-BH-A8G0 | 54 | 662 | Alive | II | Taxotere | CR | |
| 54 | TCGA-C8-A135 | 64 | 393 | Alive | II | Taxol | CR | |
| 55 | TCGA-C8-A1HM | 74 | 375 | Alive | II | Taxol | CR | |
| 56 | TCGA-C8-A27A | 48 | 747 | Alive | II | Taxol | CR | |
| 57 | TCGA-E9-A227 | 42 | 975 | Alive | II | Taxotere | CR | |
| 58 | TCGA-GM-A2DB | 62 | 2406 | Alive | II | Taxol | CR | |
| 59 | TCGA-GM-A2DC | 57 | 2535 | Alive | II | Taxotere | CR | |
| 60 | TCGA-GM-A2DF | 53 | 2155 | Alive | II | Taxol | CR | |
| 61 | TCGA-GM-A2DM | 57 | 3226 | Alive | II | Taxol | CR | |
| 62 | TCGA-GM-A3XL | 49 | 2108 | Alive | II | Paclitaxel | CR | |
| 63 | TCGA-GM-A5PV | 64 | 412 | Alive | II | Taxotere | CR | |
| 64 | TCGA-OL-A5D7 | 70 | 1780 | Alive | II | Taxotere | CR | |
| 65 | TCGA-OL-A5RU | 70 | 1219 | Alive | II | Docetaxel | CR | |
| 66 | TCGA-OL-A5RW | 40 | 1106 | Alive | II | Taxol | CR | |
| 67 | TCGA-OL-A5S0 | 66 | 620 | Alive | II | Taxol | CR | |
| 68 | TCGA-OL-A66I | 36 | 714 | Alive | II | Taxol | CR | |
| 69 | TCGA-OL-A66O | 39 | 528 | Alive | II | Taxotere | CR | |
| 70 | TCGA-OL-A66P | 75 | 428 | Alive | II | Taxotere | CR | |
| 71 | TCGA-PE-A5DE | 41 | 2645 | Alive | II | Taxotere | CR | |
| 72 | TCGA-S3-A6ZF | 64 | 502 | Alive | II | Taxotere | CR | |
| 73 | TCGA-S3-A6ZG | 61 | 399 | Alive | II | Taxol | CR | |
| 74 | TCGA-S3-AA10 | 65 | 468 | Alive | II | Taxotere | CR | |
| 75 | TCGA- S3-AA15 | 51 | None | None | II | Taxol | CR | |
| 76 | TCGA-WT-AB41 | 55 | 1611 | Alive | II | Taxotere | CR | |

Table S5 Information of breast cancer patients in TCGA database

| **No** | **Id** | **Age** | **Time(d)** | **Status** | **TNM** | **Chemotherapy** | | **Response** |
| --- | --- | --- | --- | --- | --- | --- | --- | --- |
| 77 | TCGA-A2-A3XT | 45 | 2525 | Alive | II | Docetaxel | PR | |
| 78 | TCGA-5T-A9QA | 52 | 303 | Alive | II | Taxol | CR | |
| 79 | TCGA-A8-A07I | 69 | 426 | Alive | III | Docetaxel | CR | |
| 80 | TCGA-A2-A3XU | 35 | 912 | Alive | II | Taxotere | PD | |
| 81 | TCGA-A2-A3XY | 49 | 1064 | Alive | II | Paclitaxel/Docetaxel Paclitaxel/Docetaxel | PD | |
| 82 | TCGA-E2-A1IE | 61 | 2362 | Alive | II | Paclitaxel | PD | |
| 83 | TCGA-MS-A51U | 44 | 681 | Alive | II | Paclitaxel | SD | |
| 84 | TCGA-EW-A1OY | 63 | 908 | Alive | II | Paclitaxel | SD | |
| 85 | TCGA-EW-A1P3 | 48 | 1611 | Alive | II | Paclitaxel | SD | |
| 86 | TCGA-A2-A0EW | 53 | 1884 | Death | III | Taxol | PD | |
| 87 | TCGA-A2-A3XS | 62 | 1032 | Death | III | Docetaxel | PD | |
| 88 | TCGA-AC-A2FE | 62 | 2240 | Alive | III | Taxol | PD | |
| 89 | TCGA-D8-A3Z6 | 56 | 563 | Alive | III | Taxol | SD | |
| 90 | TCGA-EW-A2FR | 59 | 1673 | Alive | III | Paclitaxel | SD | |
| 91 | TCGA-LD-A74U | 79 | 402 | Alive | III | Taxotere | SD | |
| 92 | TCGA-Z7-A8R5 | 61 | 3287 | Alive | III | Taxol | SD | |
| 93 | TCGA-LD-A7W5 | 52 | 216 | Alive | III | Taxotere | SD | |
| 94 | TCGA-S3-AA10 | 45 | 468 | Alive | IV | Docetaxel | PD | |
| 95 | TCGA-LL-A73Z | 55 | 227 | Death | IV | Taxol | PD | |

All information was downloaded in TCGA database.

CR, Complete Response.

PR, Partial Response.

SD, Stable Disease.

PD, Progressive Disease.
