# Supplementary figures and images for: Exploring Specific miRNA-mRNA Axes With Relationship to Taxanes-Resistance in Breast Cancer
Source: Front Oncol. 2020 Aug 21;10:1397. doi: 10.3389/fonc.2020.01397 (PMC7473300; doi:10.3389/fonc.2020.01397)

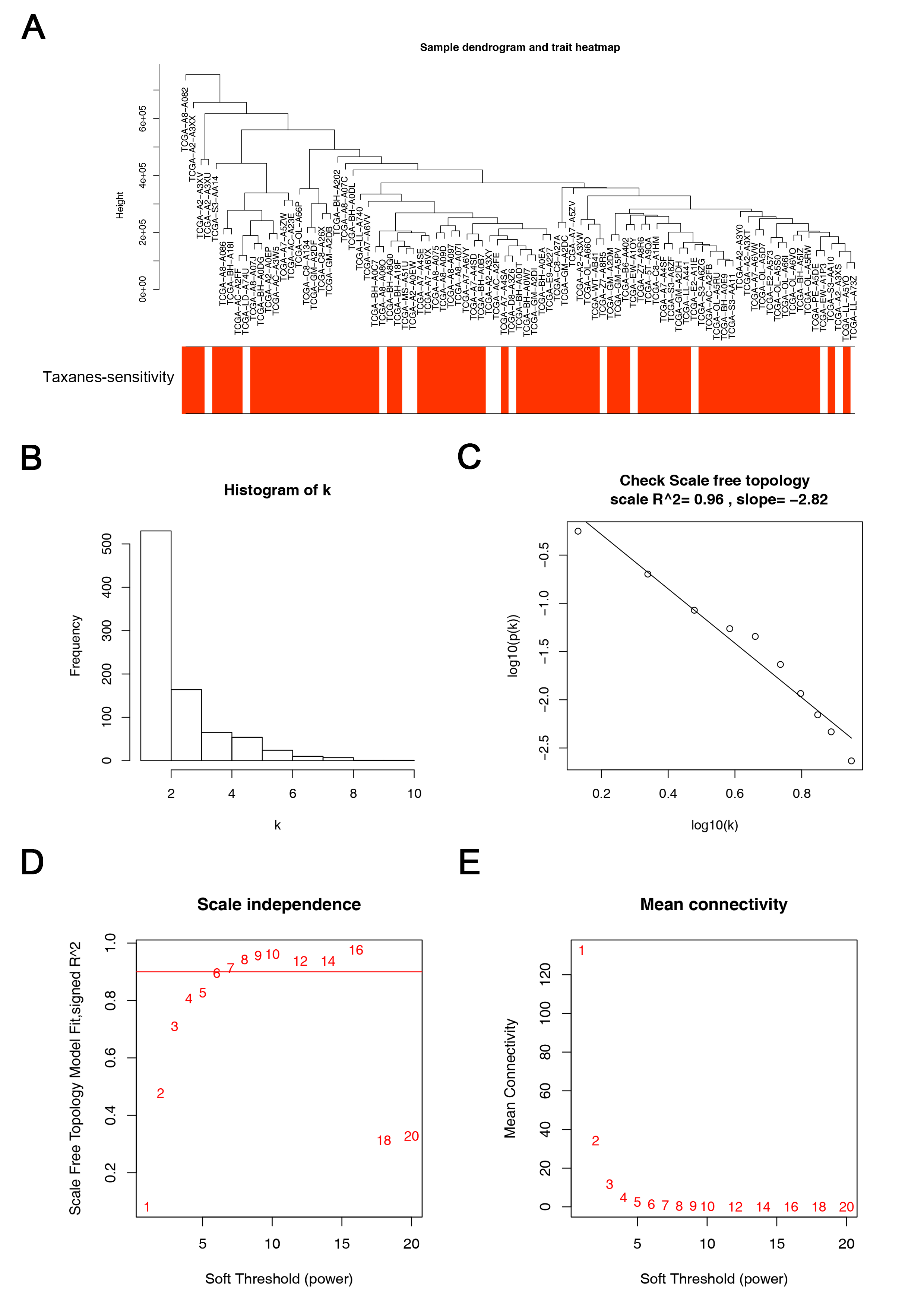

Supplement: Supplementary file 7 [file Image_1.TIF]

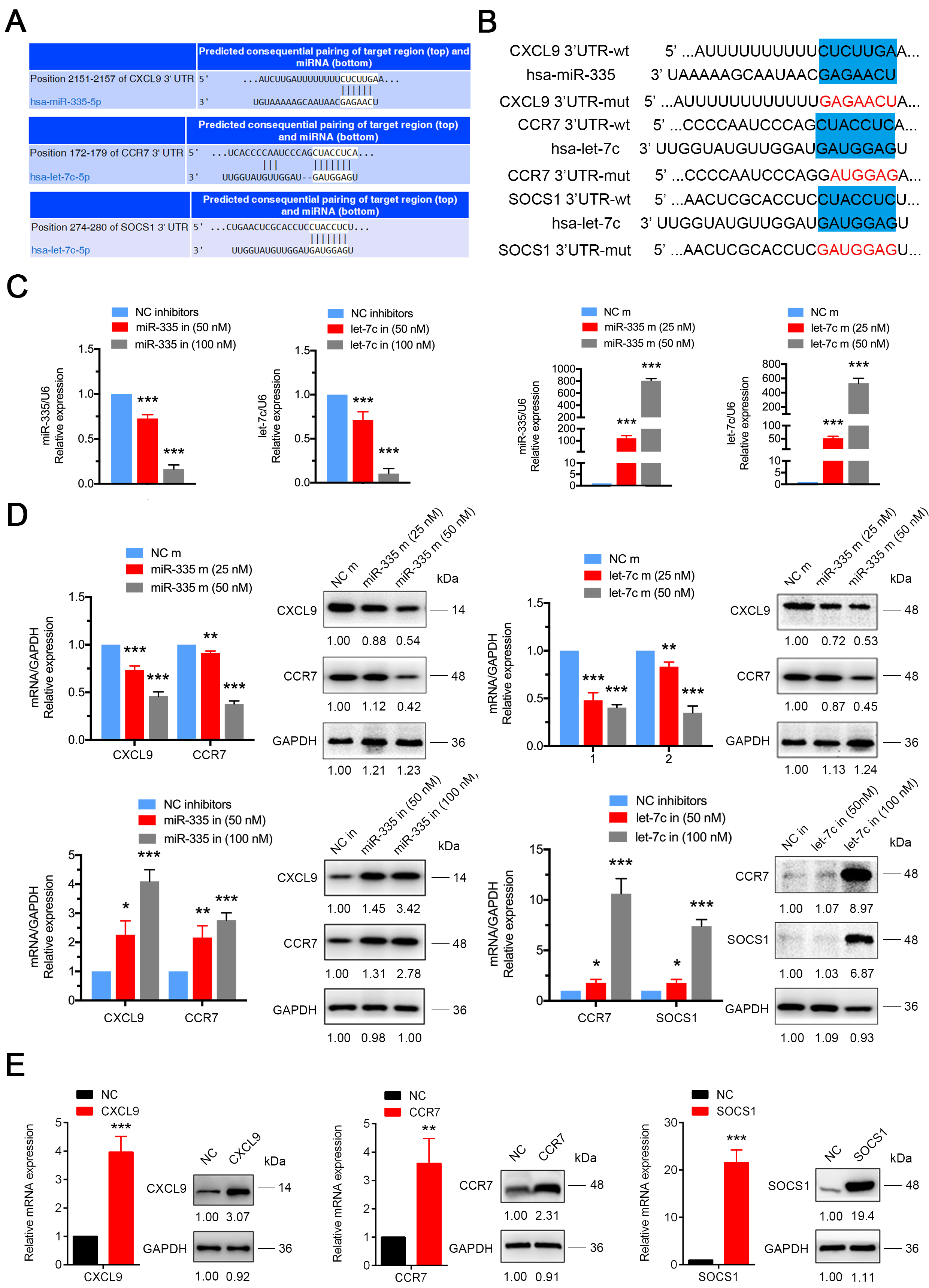

Supplement: Supplementary file 8 [file Image_2.TIF]
