# Supplementary material for: Exploring Specific miRNA-mRNA Axes With Relationship to Taxanes-Resistance in Breast Cancer
Source: Front Oncol. 2020 Aug 21;10:1397. doi: 10.3389/fonc.2020.01397 (PMC7473300; doi:10.3389/fonc.2020.01397)
Supplement: Supplementary file 10 [file Data_Sheet_1.doc]

**Supplementary Figure Legends**

Fig S1. Clustering dendrogram of breast cancer samples and the determination of soft-thresholding power.

(A) The clustering was based on differentially expressed candidate target genes relevant to therapeutic effect of taxanes. The red color represented taxanes-sensitive group, while the white color represented taxanes-resistant group.

(B) Analysis of the scale-free fit index for various soft-thresholding powers (β).

(C) Analysis of the mean connectivity for various soft-thresholding powers (β).

(D) Histogram of connectivity distribution when β = 6.

(E) Checking the scale free topology when β = 6.

Fig S2.

(A) The binding sites were predicted in TargetScan database between miR-335-5p and CXCL9, let-7c-5p and CCR7/SOCS1.

(B) The wild-type and mutant-type of 3’UTR including binding sites was cloned into the psiCHECK2 for dual-luciferase reporter assays.

(C) Quantitative PCR analysis. The transfection effect of miR-335-5p and let-7c-5p inhibitors, mimics in MCF-7/T and MCF-7 cells were respectively validated. Data was shown as mean ± SD. All assays were performed in triplicate and values represent the mean of three independent experiments.

(D) Quantitative PCR analysis and Western Blot assays. The transfection effect of miR-335 mimics/inhibitors and let-7c mimics/inhibitors were confirmed via mRNA and protein expressions of target genes.

(E) Quantitative PCR analysis and Western Blot assays. The transfection effect of plasmids overexpressing CXCL9, CCR7 and SOCS1 were validated in MCF-7/T cells.

Fig S3.

NC mimcis(50 nM)/inhibitors (100 nM)and NC control of blank plasmid (50 nM) were transfected into MCF-7/T and MCF-7 cells. Edu incorporation assays (A), colony formation assays (B), cell cycle assays (C) and cell apoptosis rate (D) were detected compared with mock cells. All experiments were performed at least three times independently. The data were presented as the mean ± SD. *P < 0.05, **P < 0.01, ***P < 0.001. Scale bar: 500 μM.
