# Supplementary material for: Exploring Specific miRNA-mRNA Axes With Relationship to Taxanes-Resistance in Breast Cancer
Source: Front Oncol. 2020 Aug 21;10:1397. doi: 10.3389/fonc.2020.01397 (PMC7473300; doi:10.3389/fonc.2020.01397)
Supplement: Supplementary file 11 [file Data_Sheet_2.docx]

**Materials and methods:**

**Breast cancer cell lines**

The human breast cell lines (HBL-100, Hs 578T, BCap37, MDA-MB-231, MDA-MB-453 and MCF-7) were purchased from the cell bank of the Chinese Scientific Academy. PTX-resistant Bads-200 cells were selected by continuous treatment of BCap37 cells for high concentrations of PTX in “time-increasing method”^[1]^. MCF-7/T were purchased from Meixuan biotechnology company, Shanghai (Table S1).

HBL-100 cells were cultured in DMEM with 10% fetal bovine serum (FBS; Biological Industries, 04-0101-1, Cromwell, CT, USA). Hs 578T cells were cultured in Dulbecco's modified Eagle's medium (DMEM; Gibco) supplemented with 0.01 mg/ml bovine insulin (Solarbio, Beijing, China) and 10% FBS. BCap37, MCF-7 cells were cultured in Roswell Park Memorial Institute (RPMI) 1640 medium (Gibco, Life Technologies, Carlsbad) with 10% FBS. MDA-MB-231 cells were cultured in Leibovitz’s L-15 medium (Gibco) with 10% FBS. Bads-200 and MCF-7/T were cultured in RPMI 1640 medium with PTX (1250 nM and 500 nM). The cell culture medium was changed every 2-3 days, and the cells were passaged with 0.25% trypsin-EDTA (Gibco, 25200056) and grown to 90% confluence. The cultures were kept at 37°C with 5% CO2 in a water-jacketed incubator (Thermo Scientific, Waltham, MA, USA). To maintain authenticity of the cell lines, frozen stocks were prepared from initial stocks, and every three months a new frozen stock was used for the experiments.

**Breast cancer patients and tissue samples**

The paraffin-embedded or fresh puncture specimen of primary breast cancer tissues were obtained at breast center, the first affiliated hospital, college of medicine, Zhejiang university. All tumor tissues were clearly diagnosed by immunohistochemistry as luminal type or triple-negative breast cancer and patients received at least two cycles of taxanes chemotherapy. Efficacy evaluation mainly included pathological and/or clinical effect evaluation. The pathological effect was evaluated according to Miller & Payne (M & P) system. The M & P pathological rating after neoadjuvant chemotherapy reached MP4 and MP5 were considered as pathologically significant groups, while MP1, MP2, and MP3 were pathologically ineffective groups. The clinical efficacy evaluation referred to the RECIST. CR and PR after neoadjuvant chemotherapy were considered to be clinically significant groups, while PD and SD as clinically ineffective group. The patients in clinically and pathologically significant group were classified as taxanes-sensitive group. The others were regarded as taxanes-resistant group. In summary, 20 patients were enrolled in taxanes-resistant group and 20 patients in taxanes-sensitive group, of luminal type breast cancer. 25 patients were enrolled in taxanes-resistant group and 25 patients in taxanes-sensitive group, of triple-negative type breast cancer. This study was approved by the Research Ethics Committee of the First Affiliated Hospital, College of Medicine, Zhejiang University. Basic information of patients was listed in Table S4.

**Dual-luciferase reporter assay.**

The 3’ UTRs of CXCL9, CCR7 and SOCS1 containing upstream miRNAs putative target sites (Fig. S2A) were amplified and cloned into psiCHECK-2 (Promega). A Fast Mutagenesis kit (VazymeBioTech) was used to mutate the binding sites as Fig. S2B according to the manufacturer’s instructions. Dual luciferase assays were performed using 1 × 10^4^ MCF-7/T cells per well in a 96-well plate. Following attachment for 12 h, the cells were co-transfected with 50 ng respective reporter constructs with either NC mimics or miRNAs (50 nM). After 48 h, the Reporter Assay System Kit (Promega) was used to measure the luciferase activity. Firefly luciferase activity was normalized to constitutiverenilla luciferase activity.

**RNA extraction, RT-PCR and quantitative real-time PCR (qRT-PCR)**

Total RNA was extracted from tissues and cell lines using RNAiso plus Reagent (TaKaRa biotechnology, Kusatsu, Japan). Total RNA was reverse transcribed into complementary DNA by using the PrimeScript RT Reageent kit (TaKaRa biotechnology, RR0037). Quantitative-PCR (q-PCR) was performed in a Roche LightCycler480 II Real-Time PCR Detection System by using TB Green Premix Ex Taq^TM^(TaKaRa biotechnology, RR420A). Quantification of miRNAs was performed with a stem-loop real-time PCR. All PCR reactions were run in triplicate, miRNAs expression relative to U6, and gene expression relative to HPRT1, were calculated using the comparative threshold method(2-ΔΔCt).

**Western blotting**

Total proteins were extracted from cells, and concentration was determined using a BCA protein assay kit (Beyotime Biotec, China). Proteins samples were fractionated using 8-10% SDS-PAGE gels and transferred to PVDF membranes (Millipore, NY, USA), after block for 1 hour with 5% non-fat milk, then incubated at 4℃ for 12 h with rabbit anti-human primary antibodies: rabbit anti-human CXCL9 (Abcam, ab9720,1:1000), CCR7 (Abcam, ab32527,1:1000) and SOCS1 (Abcam, ab3691,1:1000). Rabbit anti-human GAPDH antibodies (1:2000, Abcam) were used as endogenous controls. Protein expression levels were detected with ECL detection solution (Apexbio, Houston, USA) by G-BOX System with GeneSnap software (Syngene).

**Statistical analysis**

All experiments were performed in triplicate and the data are expressed as mean ± standard deviation (SD). Statistical analyses were performed with the GraphPad Prism Software (GraphPad). A two-tailed Student’s t-test was used to evaluate the differences between two groups of data and one-way ANOVA followed by Tukey’s posttest was used to compare the means of three independent groups. The Kaplane-Meier method and log-rank test were used to evaluate the correlation between miRNAs and gene expression and patient survival. *P < 0.05; **P < 0.01; ***P < 0.001. P values < 0.05 were considered statistically significant.

References:

1. Jiang D, Sui M, Zhong W, Huang Y, Fan W. Different administration strategies with paclitaxel induce distinct phenotypes of multidrug resistance in breast cancer cells. CANCER LETT 2013, 335(2): 404-411.
